# Attenuating Tumour Angiogenesis: A Preventive Role of Metformin against Breast Cancer

**DOI:** 10.1155/2015/592523

**Published:** 2015-03-25

**Authors:** Shan Gao, Jingcheng Jiang, Pan Li, Huijuan Song, Weiwei Wang, Chen Li, Deling Kong

**Affiliations:** Tianjin Key Laboratory of Biomaterial Research, Institute of Biomedical Engineering, Chinese Academy of Medical Science and Peking Union Medical College, Tianjin 300192, China

## Abstract

Metformin is one of the most widely prescribed antidiabetics for type 2 diabetes. A critical role of metformin against tumorigenesis has recently been implicated, although several studies also reported the lack of anticancer property of the antidiabetics. Given the controversies regarding the potential role of metformin against tumour progression, the effect of metformin against breast, cervical, and ovarian tumour cell lines was examined followed by *in vivo* assessment of metformin on tumour growth using xenograft breast cancer models. Significant inhibitory impact of metformin was observed in MCF-7, HeLa, and SKOV-3 cells, suggesting an antiproliferative property of metformin against breast, cervical, and ovarian tumour cells, respectively, with the breast tumour cells, MCF-7, being the most responsive. *In vivo* assessment was subsequently carried out, where mice with breast tumours were treated with metformin (20 mg/kg body weight) or sterile PBS solution for 15 consecutive days. No inhibition of breast tumour progression was detected. However, tumour necrosis was significantly increased in the metformin-treated group, accompanied by decreased capillary formation within the tumours. Thus, despite the lack of short-term benefit of metformin against tumour progression, a preventive role of metformin against breast cancer was implicated, which is at partially attributable to the attenuation of tumour angiogenesis.

## 1. Introduction

In recent years, epidemiological analyses have indicated a positive association between long-term diabetes and elevated risk of malignant neoplasms [1]. In particular, patients with preexisting type 2 diabetes (T2D) present a higher risk of cancer development and cancer-related mortality. Moreover, cancer patients with diabetes also showed increased mortality compared to nondiabetic cancer patients. Given the potential causal relationship between T2D and cancer, multiple plasma glucose lowering agents have been selected to be tested for potential anticancer effects, with metformin showing the most promising result.

Metformin is one of the most efficacious and safe front-line antidiabetics for type 2 diabetes (T2D). In addition to its antiglycaemic impact, recent reports also implicated critical role of metformin in tumourigenesis [1, 2]. Indeed, antiproliferative effects of metformin have been reported in multiple tumour cell lines via several molecular pathways, including the adenosine monophosphate kinase (AMPK) pathway, the insulin receptor cascade, and the AMPK-independent RagGTPase-dependent 3mTORC1 signalling network [1, 3]. Evidence also supports an anti-inflammatory role of metformin against cancer progression by inhibiting cancer stem cells [4]. In contrast, some studies observed no association between metformin and cancer-related mortality [5]. Results from a newly published epidemiological analysis also reported no direct association between metformin and cancer outcome [6]. Given the controversies regarding the use of metformin as potential anticancer treatment, we examined the effect of metformin against selective tumour cell lines followed by* in vivo* assessment of metformin on tumour growth.

## 2. Methods and Materials

### 2.1. Cell Culture and Viability Assay

Human breast (MCF-7), ovarian (SKOV-3), and cervical (HeLa) cancer cells were cultured in DMEM media (Hyclone, Beijing, China) supplemented with 10% foetal bovine serum (Gibco, Beijing, China), 100 units/mL penicillin, and 100 *μ*g/mL streptomycin (Sigma-Aldrich, Beijing, China). Cells were seeded at a density of ~5000 cells per well in 96-well plates and maintained at 37°C under standard culturing conditions. Cells were exposed to a series of concentrations of metformin (Sigma-Aldrich, Beijing, China) continuously and cell viability was determined at the end of 24 h and 5 days using a cell counting kit-8 (CCK-8; Dojindo, Japan).

### 2.2. *In Vivo* Assessment

Xenograft breast tumour models were established by injecting MCF-7 cells into 6-week-old female BALB/c nude mice (Charles River Laboratories, Beijing, China). Once the tumour size reached ~100–150 mm^3^, mice were randomly assigned to either control group or metformin-treated group. Local injection of metformin (20 mg/kg body weight) or sterile PBS was administered for 15 consecutive days. Changes of body weight were monitored and tumour volumes were measured and corrected according to standard formula [7].

### 2.3. Histomorphological and Immunofluorescence Analysis

15 days after initial injection, tumours were dissected and fixed in 4% paraformaldehyde before being paraffin embedded. Consecutive sections (thickness, 5 *μ*m) were cut onto microscope slides. Haematoxylin and eosin (H&E) staining was employed to examine tumour morphology and immunofluorescent staining using an antibody raised against von Willebrand factor (vWF; 1 : 200 dilution; Dako, Shanghai, China) was also carried out to evaluate capillary formation. The staining data were analysed with a fluorescent microscope (Leica, Germany) and fluorescent intensity was quantified using ImageJ software (National Institute of Health, USA).

## 3. Results 

### 3.1. Metformin Inhibits* In Vitro* Tumour Cell Growth

**Figure 1:**
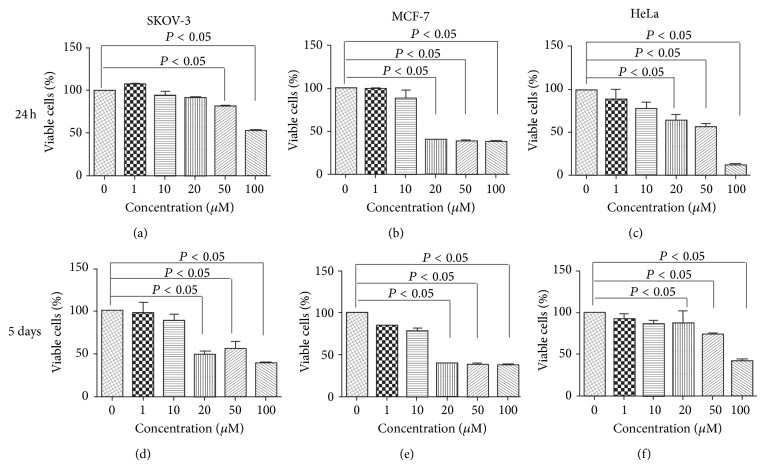
Metformin inhibits tumour cell growth. Human ovarian (SKOV-3), breast (MCF-7), and cervical (HeLa) cells were exposed to a series of concentrations of metformin for 24 h ((a), (b), and (c)) and 5 days ((d), (e), and (f)). Cell viability was assessed using a cell viability (CCK-8) assay. Data are presented as means ± SD, *n* = 6.

Given the high prevalence of ovarian, cervical, and, particularly, breast cancers in pre- and postmenopausal women, 3 female tumour cell lines, MCF-7, SKOV-3, and HeLa, were initially selected to investigate the potential anticancer effect of metformin* in vitro*. As shown in Figures 1(b) and 1(c), 24 h exposure to metformin significantly reduced cell viability in all 3 tumour cell lines, with a maximum response of 42 ± 8%, 38 ± 2%, 14 ± 2% for SKOV-3, MCF-7, and HeLa cells, respectively (Figures 1(a)–1(c)). Similar inhibitory responses were also observed from cells treated with metformin for 5 days (Figures 1(d)–1(f)). For both SKOV-3 and HeLa cells, the metformin-exerted attenuation of cell growth appeared to be concentration-dependent, in contrast to MCF-7, of which the inhibitory responses were similar once the administrative dose of metformin was over 20 mM. However, as noted by the National Cancer Institute some years ago, the activity of a pharmacological agent* in vitro* does not necessarily reflect its* in vivo* performance [8], and subsequent* in vivo* assessment was carried out.

### 3.2. Effect of Metformin on* In Vivo* Tumour Progression and Tumour Angiogenesis

Our* in vitro* cytotoxicity assay demonstrated marked inhibitory impact of metformin on ovarian, breast, and cervical cancer cell lines, with breast tumour cells, MCF-7, being the most responsive. Indeed, several studies have implicated a positive correlation of short-term use of metformin and breast carcinoma remission [9–11]. A clinical trial study also demonstrated anticancer impact of metformin in nondiabetic postmenopausal women with estrogen receptor positive breast tumours [12]. In contrast, another report observed no inhibitory benefit of metformin on multiple subtypes of breast tumours under euglycaemic condition [13], which was further supported by epidemiological studies also demonstrating a lack of anticancer property of metformin against breast carcinoma [14].

**Figure 2:**
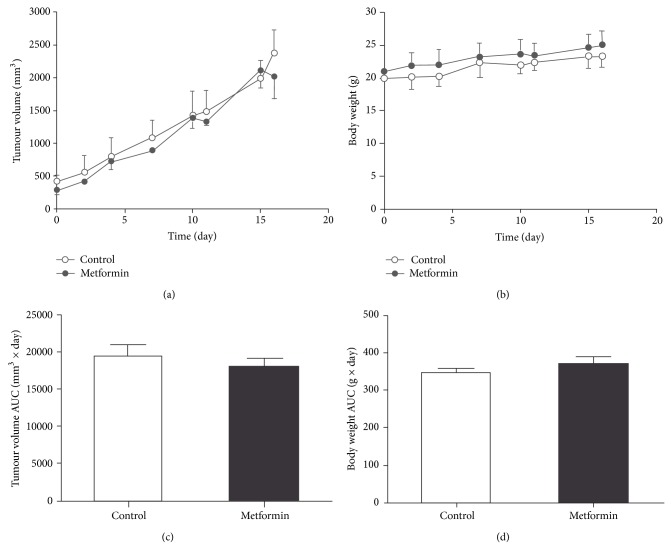
*In vivo* assessment of metformin treatment on tumour growth. Metformin (20 mg/kg body weight; Metformin group) or sterile PBS (Control group) was injected locally to mice with breast carcinoma for 15 consecutive days. (a) Average tumour size and (b) body weight were monitored and plotted against time for Metformin (closed circle) and Control group (open circle). Total changes of tumour volume (c) and body weight (d) were also presented as area under curve (AUC). Data are presented as means ± SD, *n* = 4-5.

Given the high prevalence of breast cancer and the current controversies concerning the exact impact of metformin use against breast carcinoma, human xenograft breast tumour mouse models were used in the present study for* in vivo* evaluation. Thus, local injection of PBS (Control group) or metformin (20 mg/kg body weight; Metformin group) was administered daily at the tumour site for two weeks. No changes of tumour volume were detectable between the control and metformin-treated groups (Figures 2(a) and 2(b)). No attenuation of tumour progression was observed either as superimposable tumour growth profiles were obtained from both groups (Figure 2(a)). In addition, no significant difference in animal body weight was detected between the two groups (Figure 2(b)).

**Figure 3:**
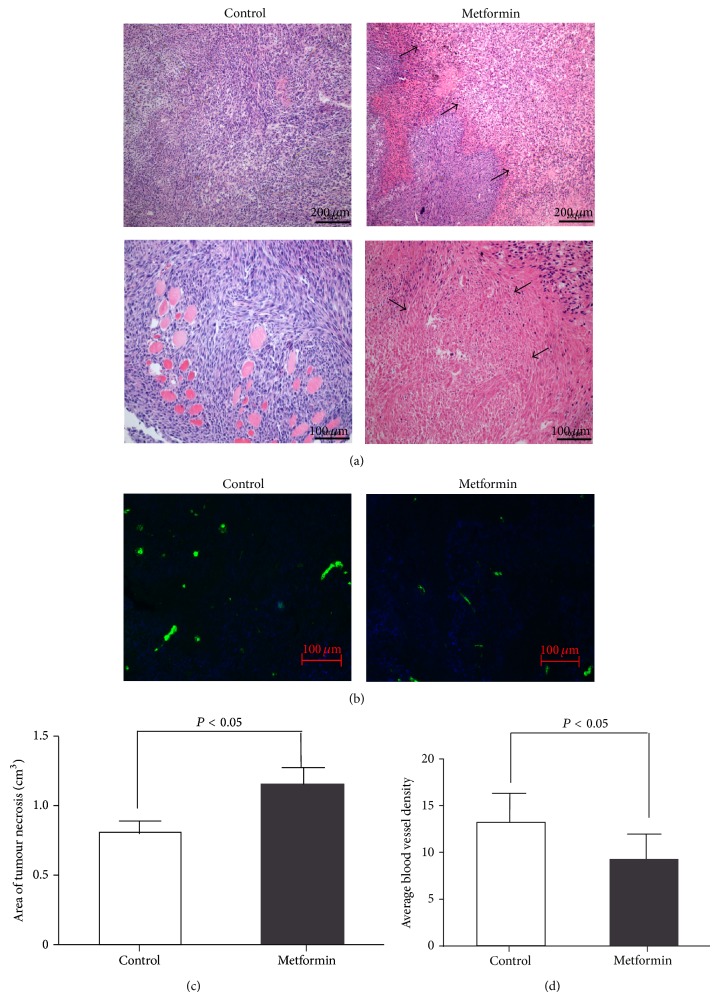
Effect of metformin treatment on tumour necrosis and angiogenesis. H&E staining of tumours obtained from Metformin and Control groups. Scale bar: 200 *μ*m ((a), upper panels) and 100 *μ*m ((a), lower panels). (b) Immunofluorescent staining of vWF (green) in tumours obtained from Metformin and Control groups. Scale bar: 100 *μ*m. Nuclei were counter-stained with DAPI (blue). Area of tumour necrosis (c) and tumour blood vessel density (d) were quantified. Tumour necrosis was indicated by black arrows. Data are presented as means ± SD, *n* = 4-5. Images are representative of 4-5 animals from 3 separate experiments.

Despite the lack of inhibitory impact of metformin on tumour growth, subsequent histological analyses revealed marked increase of tumour necrosis in metformin-treated mice (143 ± 11% over control group, *P* < 0.01; Figures 3(a) and 3(c)). Furthermore, immunofluorescence staining of von Willebrand factor (vWF), a microvascular endothelial marker, also revealed reduced average blood vessel density in tumours obtained from the metformin-treated animals (69 ± 29% over control group, *P* < 0.02; Figures 3(b) and 3(d)), implicating an antiangiogenic impact of metformin.

## 4. Discussion

The cytotoxicity of metformin was observed in all 3 cell types, with breast tumour cells being the most responsive, although cautions need to be exercised when drawing conclusions from* in vitro* results since cultured tumour cells are morphologically and functionally different from native tumours.

Subsequent* in vivo* assessment showed no detectable tumour reduction after local injection of metformin (20 mg/kg body weight) for 15 days. In fact, the effect of metformin against breast cancer has long been extensively investigated albeit contradictory as summarized in a recent review [2]. Most studies have reported decreased incidents and severity of mammary cancer in rodent models after long-term oral or intravenous administration of metformin. Similarly, attenuated tumour progression was observed in humans following treatment with high dosage metformin [2]. In contrast, no inhibition of tumour growth and latency was also recorded, often when low dosage of metformin was applied. Considering the potential implications of different dosage and administrative routes of metformin treatment on cancer outcome, 20 mg per kg body weight metformin was used in the present study and the drug was directly injected to the tumour sites to minimize non-tumour-site distribution caused by different routes of administration [2]. We observed no attenuation of tumour growth after short-term administration of a moderate level of metformin, which suggests limited short-term anticancer ability of metformin treatment* per se*. However, this result may not reflect long-term effect of the drug as the necrosis area was considerably larger in tumours obtained from metformin-treated mice.

In addition, significant attenuation of capillary formation was also evident from the metformin-treated group, consistent with a previous report proposing an AMPK/mTOR-dependent antiangiogenic effect of metformin on ovarian cancer [15]. Thus, despite the lack of short-term benefit of metformin in tumour regression* in vivo*, a preventive role of metformin against breast cancer was implicated, which is at least partially attributable to the attenuation of tumour angiogenesis. Further investigation is required to evaluate whether the antiangiogenic effect of metformin is tumour-specific, particularly since metformin is widely prescribed as an antidiabetic and T2D patients have an elevated risk of vascular disorders. Furthermore, considering the diversity of metformin action, the exact mechanisms underlying the antiangiogenic property of metformin are also required to be elucidated.
